# Assessment of hematologic indices and their correlation to hemoglobin A1c among Bosnian children with type 1 diabetes mellitus and their healthy peers

**DOI:** 10.5937/jomb0-25315

**Published:** 2021-03-12

**Authors:** Suzana Tihić-Kapidžić, Adlija Čaušević, Jasmina Fočo-Solak, Maja Malenica, Tanja Dujić, Sniježana Hasanbegović, Nermina Babić, Ermin Begović

**Affiliations:** 1 University of Sarajevo, Clinical Centre, Department for Clinical Biochemistry and Immunology, Sarajevo, Bosnia and Herzegovina; 2 University of Sarajevo, Faculty of Pharmacy, Department of Biochemistry and Clinical Analysis, Sarajevo, Bosnia and Herzegovina; 3 Clinical Centre University of Sarajevo, Pediatric Clinic, Sarajevo, Bosnia and Herzegovina; 4 University of Sarajevo, Medical Faculty, Department of Human Physiology, Sarajevo, Bosnia and Herzegovina

**Keywords:** hematological indices, hemoglobin A1c, type 1 diabetes mellitus, pediatric population, hematološki parametri, hemoglobin A1c, dijabetes melitus tip 1, deca

## Abstract

**Background:**

Altered levels of many hematological parameters have been directly associated with diabetes in adults, while studies on children with type 1 diabetes mellitus are lacking. The aim of this study was to determine hematological indices in diabetic Bosnian children in comparison to healthy controls as well as to correlate their levels to blood glucose and hemoglobin A1c.

**Methods:**

100 healthy and 100 children with type 1 diabetes mellitus (age 1-18) were included in this study. Complete blood count, hemoglobin A1c, and glucose were tested. Results were analysed by IBM SPSS Statistics version 23.

**Results:**

Significant differences (p<0.05) between healthy and diabetic children were found in relation to HbA1c, glucose, mean platelet volume, the number of white blood cells and erythrocytes, hematocrit, hemoglobin and MCH values. No gender differences or significant age differences were seen for hemoglobin, hematocrit, and MCV, while platelets, MPV, and MCH differed by age only in healthy children. When diabetic children were classified according to HbA1c levels, significant differences were seen for erythrocyte count and hematocrit value (p=0.013 and 0.019, respectively). The number of erythrocytes and white blood cells correlated significantly with HbA1c (p=0.037 and 0.027, respectively).

**Conclusions:**

Lower levels of erythrocytes, hematocrit, and hemoglobin in diabetic compared to healthy children indicate possible development of anemia, while higher MCV, MCH, and MPV values indicate an alteration in erythrocyte morphology. Hematological indices could be a useful inexpensive tool in the diagnosis and follow up of type 1 diabetes in children.

## Abstract (sr)

**Uvod:**

Brojne studije ukazuju na promene hematološkog statusa kod odrasle populacije sa dijabetesom, dok su istraživanja na deci sa tipom 1 dijabetes melitusa nedovoljna. Cilj ovog istraživanja je bio utvrditi hematološki status kod dijabetične bosanske dece i uporediti ga sa zdravom kontrolnom grupom te analizirati vezu hematoloških parametara sa koncentracijama glukoze i hemoglobina A1c u serumu.

**Metode:**

U istraživanje je uključeno 100 zdrave i 100 dece sa tipom 1 dijabetes melitusa, starosti od 1 do 18 godina. Kompletna krvna slika, hemoglobin A1c i glukoza su testirani kod sve dece. Analiza podataka je urađena pomoću programa IBM SPSS Statistics, verzija 23.

**Rezultati:**

Značajne razlike (p<0,05) između zdrave i dijabetične dece su uočene u vrednostima HbA1c, glukoze, MPV, broju leukocita i eritrocita, hematokrita, hemoglobina i MCH. Polne razlike u vrednostima ispitivanih parametara nisu nađene. Starosne razlike su bile značajne za vrednosti hemoglobina, hematokrita i MCV. Broj trombocita, MPV i MCH su pokazali starosne razlike samo kod zdrave dece. Podela dijabetične dece prema vrednostima HbA1c je pokazala da postoje značajne razlike samo u broju eritrocita i vrednosti hematokrita (p=0,013 i 0,019, respektivno). Broj eritrocita i leukocita je pokazao statistički značajne pozitivne korelacije sa HbA1c (p=0,037 i 0,027, respektivno).

**Zaključak:**

Niže vrednosti eritrocita, hematokrita i hemoglobina kod dijabetične u odnosu na zdravu decu indiciraju potencijalni razvoj anemije, dok više vrednosti MCV, MCH i MPV ukazuju na morfološke promene eritrocita. Hematološki parametri mogu biti jeftini i korisni pokazatelji u dijagnozi i praćenju dece sa tipom 1 dijabetes melitusa.

## Introduction

Type 1 diabetes mellitus (T1DM) is one of the most common endocrinological disorders in children and adolescents [1] [2]. It is caused by various environmental factors interacting with an underlying genetic predisposition resulting in autoimmune destruction of pancreatic beta cells [3] [4]. The onset of type 1 diabetes mellitus occurs early in life and children affected are at great risk of developing microvascular and macrovascular complications and cardiovascular disease later as a complication of diabetes. Hyperglycemia, dyslipidemia, inflammation, and stress are factors that increase the risk of vascular complications in patients with T1DM [2] [5]. Those long-term complications are the leading cause of premature mortality in this group of patients [6] [7] [8]. T1DM complications that develop in children and adolescents depend on disease duration, metabolic control level, environmental and genetic factors.

T1DM and its complications represent a heavy financial burden on diabetic patients, their families, and society. This is especially true in developing countries. Hematological indices from complete blood counts may be used to overcome these challenges as good indicators and independent predictors for various microvascular and macrovascular complications related to endothelial dysfunction and inflammation. Altered level of many hematological parameters such as red blood cells (RBCs), white blood cells (WBC), and the platelet function and morphology have been observed and shown to be directly associated with diabetes [1] [2] [3] [4] [9] [10] [11] [12] [13] [14] [15] [16] [17] [18] and metabolic syndrome in the adult population, indicating their close relationship with different components of metabolic syndrome, including insulin resistance [19] [20] [21] [9] [10]. These studies demonstrated a significant difference in red blood cell distribution width as well as platelet volume between type 2 diabetic patients and controls (both increased in diabetic patients) [19] [20] [21]. Differences also manifested at the level of total white blood cells, absolute lymphocyte count, absolute neutrophil count when compared to controls (all three increased significantly in diabetic patients) [20].

Biadgio et al. [20], found that among platelet indices, mean platelet volume, and platelet distribu-tion width were significantly increased in diabetic patients. In their study, some of the parameters of complete blood count (CBC) were found to be useful tools in following type 2 diabetes and its complications.

Milosevic et al. [21] reported independent associations of hematological parameters with insulin resistance and glycemic control. This association was also reported by Uko et al. [22] in type 1 diabetic patients, where changes in packed cell volume, platelet, and total white cell count as well as anemia, thrombocytosis and leukocytosis were reported.

Studies related to the issue of diagnostic significance of different hematological markers in children diabetic population are lacking worldwide due to unrecognized steep rise in the incidence of childhood diabetes affecting all countries in the world, including Bosnia and Herzegovina. Therefore, the aim of this study was to determine hematological indices (RBC count, hemoglobin, hematocrit, MCV, MCH, MCHC, RDW, PLT count, platelet morphology, platelet activation indicator MPV) in Bosnian children with T1DM and compare them to healthy controls of the same age as well as to correlate their levels to blood glucose, HbA1c levels, and anthropometric measurements. Of particular interest was to compare the sex and age differences in the examined parameters. To our knowledge, this is the first study of this kind in Bosnia and Herzegovina.

## Materials and Methods

### Patient population and study design

Two hundred children aged between 1-18 years were included in this study. The whole study group (N=200) consisted of 100 (50%) healthy children and 100 (50%) children with type 1 diabetes mellitus treated with insulin at the Department of Paediatrics, University Clinical Centre Sarajevo. The diabetic group was at the later phase divided into three groups according to HbA1c levels.

The control group was age-and sex-matched to the study group. The mean age of children enrolled in the study was 10.36±3.85 years. In a group with T1DM, 13 (13%) children were 1-6 years old, 58 (58%) were 7-12 years old, and 29 (29%) 13-18 years old. In the healthy control group, 15 (15%) children were 1-6 years old, 58 (58%) were 7-12 years old, and 27 (27%) 13-18 years old. The male to female ratio in the control group was 1.2, and 1.0 in children with T1DM. The group of children with T1DM consisted of 50 (50%) male and 50 (50%) female subjects, while the control group consisted of 55 (55%) male and 45 (45%) female.

Written informed consent was obtained from all participants recruited into this study (controls and diabetic patients). The study was approved by the Ethical Committee of the University Clinical Centre Sarajevo and was carried out in compliance with the Helsinki Declaration.

After obtaining detailed histories for all the children involved in the study, they were physically examined by a medical doctor (MD), and data were recorded on forms prepared for this study. Collected data included age (year), sex, height (cm), weight (kg), body mass index (BMI) (kg/m^2^), family history of chronic diseases, and the laboratory findings. Patients with a history of accompanying chronic disease, current acute infection, alcohol abuse, and atherosclerotic disease or with severe hypoglycemia or diabetic ketoacidosis were excluded from the study.

### Laboratory tests

The following laboratory tests were performed on healthy and children with T1DM: white blood cell count (10^12^/L), red blood cell count (10^12^/L), hemoglobin (g/L), hematocrit (%), MCV (fL), MCH (pg), MCHC (g/L), RDW (%CV), platelet count (10^9^/L), MPV (fL), HbA1c (%), glucose (mmol/L). Blood samples were taken from the brachial vein after overnight fasting and were processed within 2 hours after collection, according to the standard operating procedure of our institution. Glucose and HbA1c levels were acquired from blood samples that were centrifuged in gel tubes (3000 rpm for 5 minutes). Red blood cell and platelet morphology parameters were extracted from routinely performed complete blood count results. The blood counts were performed on Cell-Dyn Sapphire (Abbott Diagnostic, USA). Glucose levels were determined with Architect ci8200 (Abbott Diagnostics, USA), and HbA1c was measured using HPLC method on Adams analyzer (Arkray, USA).

### Statistical analysis

Statistical evaluation of results was performed by IBM SPSS Statistics version 23 (Chicago, IL, USA). Median, interquartile range (IQR), ratio, and frequency values were used for the descriptive statistics of the data. Data distribution was tested using the Kolmo-gorov-Smirnov test. The parameters were compared within two groups using the Mann-Whitney U test. Spearman's rho correlation analysis was used to assess the relationships between the parameters. The Kruskal-Wallis test was used for the comparison of multiple groups. P-value <0.05 was considered statistically significant.

## Results

There were no differences in age, BMI, total platelet count, MCV, MCHC, and RDW between healthy and children with T1DM (Table 1). The Mann-Whitney U test showed a significant difference between the healthy control group and children with T1DM in HbA1c, glucose, MPV, number of white blood cells, number of erythrocytes, hematocrit and hemoglobin values, with p<0.001 for all 7 parameters (Table 1). MCH also shows a significant difference between healthy and children with T1DM, with p=0.035 (Table 1).

**Table 1:** Anthropometric, clinical and hematological characteristics of children with type 1 diabetes mellitus and healthy controls Values represent medians (lower-upper quartile), p – significance between healthy controls and children with type 1 diabetes. All differences were tested using the Mann-Whitney U test.BMI, body mass index; HbA1c, hemoglobin A1c; MPV, mean platelet volume; RBC, red blood cells; MCV, mean corpuscular volume; MCH, mean corpuscular hemoglobin; MCHC, mean corpuscular hemoglobin concentration; RDW, red blood cell distribution width; WBC, white blood cells.

Parameter	Healthy Controls (N=100)	Type 1 diabetes (N=100)	p
Age (years)	9.0 (8.0–13.0)	10.5 (8.0–14.0)	0.071
BMI (kg/m^2^)	18.5 (15.8–21.3)	18.4 (16.3–20.2)	0.920
HbA1c (%)	5.1 (4.9–5.2)	7.9 (7.0–8.7)	<0.001
Platelets (10^9^/L)	286.5 (253.5–330.8)	274.5 (235.3–326.8)	0.141
MPV (fL)	6.3 (5.6–6.8)	7.5 (6.5–9.3)	<0.001
Glucose (mmol/L)	4.7 (4.4–5.0)	13.5 (9.3–18.3)	<0.001
RBC (10^12^/L)	5.2 (4.9–5.4)	4.9 (4.6–5.1)	<0.001
Hemoglobin (g/L)	143.0 (138.0–148.0)	137.0 (129.3–143.8)	<0.001
Hematocrit (%)	43.3 (41.4–45.1)	40.7 (39.1–43.7)	<0.001
MCV (fL)	82.9 (81.3–85.7)	83.4 (80.6–88.3)	0.476
MCH (pg)	27.5 (26.8–28.5)	28.1 (27.0–29.2)	0.035
MCHC (g/L)	330.0 (327.0–336.0)	332.5 (320.0–348.0)	0.514
RDW (%CV)	11.8 (11.4–12.1)	11.6 (10.6–12.4)	0.088
WBC (10^12^/L)	7.3 (5.9–8.7)	6.1 (4.9–7.5)	<0.001

When healthy and diabetic populations were divided according to gender, statistically significant gender differences were observed only at the level erythrocyte count, hemoglobin, and MCV (p=0.007, 0.048, and 0.012) in the group of diabetic patients (Table 2). Healthy controls showed no gender differences in any of the examined parameters (Table 2).

**Table 2:** Gender differences in anthropometric, clinical and hematological characteristics of children with type 1 diabetes mellitus and healthy controls Values represent medians (lower-upper quartile), p* – significance between males and females in the group of healthy controls. p** – significance between males and females in children with type 1 diabetes. All differences were tested using the Mann-Whitney U test. BMI, body mass index; HbA1c, hemoglobin A1c; MPV, mean platelet volume; RBC, red blood cells; MCV, mean corpuscular volume; MCH, mean corpuscular hemoglobin; MCHC, mean corpuscular hemoglobin concentration; RDW, red blood cell distribution width; WBC, white blood cells.

Parameter	Healthy Controls			Type 1 Diabetes		
	Male (N=55)	Female (N=45)	p*	Male (N=50)	Female (N=50)	p**
Age (years)	9.0 (7.0–13.0)	9.0 (8.0–13.0)	0.564	10.0 (7.8–12.0)	12.0 (9.0–15.0)	0.097
BMI (kg/m^2^)	18.6 (16.4–20.4)	17.4 (15.4–21.6)	0.716	16.3–19.4	18.5 (16.4–21.0)	0.182
HbA1c (%)	5.2 (4.9–5.3)	5.1 (4.9–5.2)	0309	7.9 (7.0–8.7)	7.9 (7.1–8.5)	0.738
Platelets (10^9^/L)	293.0 (255.0–334.0)	283.0 (251.5–329.0)	0.554	272.5 (229.3–315.8)	276.0 (239.3–339.8)	0.340
MPV (fL)	6.3 (5.8–6.8)	6.2 (5.5–6.9)	0.371	7.4 (6.5–9.5)	7.6 (6.4–9.2)	0.775
Glucose (mmol/L)	4.7 (4.5–5.1)	4.7 (4.4–4.9)	0.420	13.1 (8.7–18.9)	14.4 (10.0–17.3)	0.863
RBC (10^12^/L)	5.3 (5.15.4)	5.1 (4.9–5.4)	0.190	5.0 (4.8–5.3)	4.7 (4.5–5.0)	0.007
Hemoglobin (g/L)	144.0 (138.0–149.0)	143.0 (138.0–147.5)	0.739	140.5 (130.8–144.3)	135.5 (129.0–141.0)	0.048
Hematocrit (%)	43.2 (41.4–45.1)	43.3 (41.5–45.1)	0.816	40.8 (39.3–44.1)	40.4 (38.9–43.1)	0.377
MCV (fL)	82.9 (81.3–85.6)	83.4 (81.3–86.2)	0.425	81.9 (79.6–86.8)	85.2 (81.5–90.7)	0.012
MCH (pg)	27.5 (26.8–28.4)	27.7 (26.9–29.0)	0.427	27.8 (26.9–28.9)	28.3 (27.4–29.8)	0.224
MCHC (g/L)	332.0 (326.0–336.0)	330.0 (327.5–336.0)	0.920	338.0 (321.8–349.0)	329.0 (319.0–345.0)	0.177
RDW (%CV)	11.8 (11.5–12.1)	11.7 (11.4–12.1)	0.474	11.7 (10.5–12.3)	11.5 (10.7–12.4)	0.823
WBC (10^12^/L)	7.3 (6.2–8.5)	7.3 (5.7–9.3)	0.724	5.9 (4.8–7.5)	6.4 (5.2–7.9)	0.528

In the analysis of the study populations according to their age, hemoglobin, hematocrit, and MCV showed statistically significant differences between compared age groups, both in diabetic children and their healthy peers (Table 3). MCHC and RDW were significantly different within compared age groups in diabetic children (p=0.02 and p=0.007, respectively), while platelets, MPV, and MCH showed statistically significant age differences in healthy children (p=0.06, p=0.06, and p=0.01, respectively) (Table 3). There were no other statistically significant age differences within the study groups except for those expected, such as age and BMI (Table 3).

**Table 3:** Age differences in anthropometric, and hematological characteristics of children with type 1 diabetes mellitus and healthy controls Values represent medians (lower-upper quartile), p* – significance between age groups in healthy controls. p** – significance between age groups in children with type 1 diabetes. All differences were tested using the Kruskal-Wallis test. BMI, body mass index; HbA1c, hemoglobin A1c; MPV, mean platelet volume; RBC, red blood cells; MCV, mean corpuscular volume; MCH, mean corpuscular hemoglobin; MCHC, mean corpuscular hemoglobin concentration; RDW, red blood cell distribution width; WBC, white blood cells.

Parameter	Healthy Controls				Type 1 Diabetes			
	1–6 years (N=15)	7–12 years (N=58)	13–18 years (N=27)	p*	1–6 years (N=13)	7–12 years (N=58)	13–18 years (N=29)	p**
Age (years)	4.0 (3.0–5.0)	9.0 (8.0–10.3)	14.0 (13.0–16.0)	<0.001	5.0 (3.5–6.0)	10.0 (9.0–12.0)	16.0 (14.0–17.0)	<0.001
BMI (kg/m^2^)	15.2 (14.8–16.4)	18.4 (15.4–20.2)	21.4 (18.6–23.2)	<0.001	18.1 (15.9–19.3)	17.2 (15.8–18.6)	21.0 (19.3–23.4)	<0.001
HbA1c (%)	5.1 (4.9–5.2)	5.1 (4.9–5.2)	5.1 (4.9–5.3)	0.928	8.2 (7.8–8.8)	7.7 (6.8–8.6)	8.0 (7.3–8.9)	0.656
Platelets (10^9^/L)	297.0 (259.0–327.0)	304.0 (274.8–337.3)	253.0 (242.0–288.0)	0.06	294.0 (247.5–359.5)	264.5 (223.0–314.0)	283.0 (235.5–343.5)	0.299
MPV (fL)	6.2 (5.5–6.8)	6.1 (5.5–6.6)	6.8 (6.1–7.5)	0.06	7.2 (6.2–7.8)	7.4 (6.4–9.2)	7.9 (7.0–10.2)	0.675
Glucose (mmol/L)	4.6 (4.0–4.7)	4.9 (4.4–5.1)	4.8 (4.4–5.0)	0.08	11.6 (10.5–18.7)	14.1 (8.9–19.0)	11.7 (7.8–16.9)	0.502
RBC (10^12^/L)	5.2 (4.9–5.3)	5.2 (4.9–5.4)	5.3 (5.1–5.6)	0.136	4.8 (4.5–5.1)	4.9 (4.6–5.1)	4.9 (4.6–5.2)	0.491
Hemoglobin (g/L)	138.0 (131.0–142.0)	143.0 (136.8–147.0)	149.0 (146.0–156.0)	<0.001	129.0 (126.5–139.5)	136.0 (129.8–144.0)	141.0 (135.5–143.5)	0.05
Hematocrit (%)	41.2 (39.7–42.8)	42.9 (41.3–44.5)	45.1 (43.4–46.8)	<0.001	39.2 (36.9–39.6)	40.6 (38.9–43.1)	43.0 (40.6–46.7)	<0.001
MCV (fL)	80.9 (80.0–82.2)	82.9 (81.2–85.5)	85.7 (82.9–88.4)	<0.001	79.2 (77.1–81.5)	82.4 (80.4– 86.5)	88.4 (85.3–92.4)	<0.001
MCH (pg)	27.0 (26.4–27.7)	27.5 (26.8–28.4)	28.2 (27.4–29.3)	0.01	27.7 (25.7–29.0)	27.9 (27.0– 28.9)	28.3 (27.5–30.1)	0.481
MCHC (g/L)	333.0 (327.0–336.0)	330.0 (327.0–335.3)	332.0 (324.0–338.0)	0.592	343.0 (327.5–366.5)	337.0 (323.8–349.3)	320.0 (308.0–335.5)	0.020
RDW (%CV)	11.8 (11.6–11.9)	11.7 (11.4–12.0)	11.9 (11.3–12.4)	0.467	11.1 (10.2–12.1)	11.5 (10.3–12.1)	11.8 (11.2–15.1)	0.007
WBC (10^12^/L)	7.3 (5.3–9.6)	7.2 (5.9–8.4)	7.5 (6.4–8.7)	0.776	7.1 (5.9–8.7)	5.9 (4.8–7.4)	5.9 (5.2–8.1)	0.126

In the next phase, the effects of glycaemia control were tested in the population of diabetic children. After baseline evaluation, diabetic patients were divided into three groups according to their HbA1c levels: Group A consisted of patients with HbA1c levels <7%, group B consisted of patients with HbA1c levels between 7.1-8.4 and group C with HbA1c 8.5% (Table 4). The latest blood A1C targets for children and adolescents with type 1 diabetes, according to the American Diabetic Association 2019 criteria [12] is 7.5%. A lower goal (<7.0%) is reasonable if it can be achieved without excessive hypoglycemia.

**Table 4:** Type 1 diabetes mellitus patients grouped by levels of HbA1c Values represent frequency, number of children in each group determined by values of HbA1c (hemoglobin A1c).

	HbA1c <7%	HbA1c 7.1–8.4%	HbA1c >8.5%	Total
Healthy Controls	100	0	0	100
Type 1 diabetes	26	44	30	100
Total	126	44	30	200

All of the healthy children in the control group had HbA1c values <6% (Table 4). According to HbA1c values, 26% of children had an excellent level of disease control, 44% of children showed a somewhat worse level of disease control, while 30% of children with T1DM had essentially uncontrolled disease with HbA1c levels reaching 15% or more (Table 4).

No significant differences were observed at the level of age, BMI, WBC, hemoglobin, MCV, MCH, MCHC, RDW, PLT count, and MPV between compared groups (Table 5). Statistically significant differences between groups were observed only for erythrocyte count and hematocrit value (p=0.013 and 0.019, respectively), in addition to expected differences in glucose and HbA1c levels (Table 5).

**Table 5:** Influence of glycemia control on the values of anthropometric and hematological parameters in children with type 1 diabetes Values represent medians (lower-upper quartile), p – significance between HbA1c level groups in diabetic children. All differences were tested using the Kruskal-Wallis test. BMI, body mass index; HbA1c, hemoglobin A1c; MPV, mean platelet volume; RBC, red blood cells; MCV, mean corpuscular volume; MCH, mean corpuscular hemoglobin; MCHC, mean corpuscular hemoglobin concentration; RDW, red blood cell distribution width; WBC, white blood cells.

Parameter	HbA1c <7%(N=26)	HbA1c 7.1–8.4% (N=44)	HbA1c >8.5% (N=30)	p
Age (years)	10.0 (8.8–12.0)	10.5 (8.0–15.0)	11.5 (8.8–13.3)	0.886
BMI (kg/m^2^)	17.2 (15.7–18.9)	18.5 (16.4–20.8)	18.4 (16.9–20.3)	0.362
HbA1c (%)	6.6 (6.1–6.9)	7.9 (7.4–8.2)	9.3 (8.7–10.5)	<0.001
Platelets (10^9^/L)	261.0 (229.3–307.3)	272.0 (234.0–335.0)	288.0 (239.8–358.8)	0.181
MPV (fL)	7.4 (6.5–8.9)	7.6 (6.7–9.3)	7.5 (6.1–9.4)	0.316
Glucose (mmol/L)	11.2 (6.1–14.5)	12.7 (9.7–16.6)	18.6 (11.5–21.5)	<0.001
RBC (10^12^/L)	4.6 (4.4–5.1)	4.9 (4.7–5.1)	4.9 (4.7–5.3)	0.013
Hemoglobin (g/L)	134.0 (125.8–144.0)	140.0 (131.0–143.0)	137.0 (131.5–144.0)	0.276
Hematocrit (%)	40.1 (37.8–41.3)	41.0 (39.5–44.3)	41.3 (39.0–44.4)	0.019
MCV (fL)	83.9 (81.0–87.5)	84.7 (79.9–89.5)	81.8 (80.1–87.4)	0.730
MCH (pg)	28.7 (27.5–29.8)	28.1 (26.8–28.9)	27.7 (26.9–29.2)	0.114
MCHC (g/L)	343.0 (329.0–358.0)	328.0 (315.3–340.5)	333.5 (319.8–346.3)	0.183
RDW (%CV)	11.6 (10.5 –12.1)	11.6 (10.8–12.9)	11.4 (10.5–12.6)	0.752
WBC (10^12^/L)	5.6 (4.8–6.9)	5.9 (4.9–7.9)	6.9 (5.7–7.9)	0.067

Spearman correlation analysis (Table 6) showed significant positive correlations between BMI, HGB, HCT, MCV, RDW, and age in diabetic Bosnian children. MCHC showed a significant negative correlation with age. MPV showed significant negative correlations with platelet and leukocyte counts, and significant positive correlations with hematocrit and RDW. The number of erythrocytes, as well as white blood cells, showed a significant positive correlation with HbA1c. Other correlations of hematological indices with HbA1c and glucose in diabetic children were not significant. Spearman correlation between erythrocyte and HbA1c values showed a significant positive correlation (Spearman rho=0.208 with p=0.037). Spear man correlation between white blood cells and HbA1c values showed a significant negative correlation (Spearman rho=0.222 with p=0.027).

**Table 6:** Spearman correlation for analysed parameters in children with type 1 diabetes mellitus (N=100) All correlations were tested using Spearman test. Spearman ρ (rho), value of Spearman’s rank correlation coefficient, where »-« represents negative correlation, »**« correlation is significant at the 0.01 level, »*« correlation is significant at the 0.05 level. p – significance of correlation between parameters. BMI (kg/m^2^), body mass index; HbA1c (%), hemoglobin A1c; PLT (10^9^/L), platelets; MPV (fL), mean platelet volume; GLU (mmol/L), glucose; RBC (10^12^/L), red blood cells; HGB (g/L), hemoglobin; HCT (%), hematocrit; MCV (fL), mean corpuscular volume; MCH (pg), mean corpuscular hemoglobin; MCHC (g/L), mean corpuscular hemoglobin concentration; RDW (%CV), red blood cell distribution width; WBC (10^12^/L), white blood cells.

		AGE	BMI	HbA1c	PLT	MPV	GLU	RBC	HGB	HCT	MCV	MCH	MCHC	RDW
BMI	Spearman ρ (rho)	.496**	/	/	/	/	/	/	/	/	/	/	/	/
	p	<0.001												
HbA1c	Spearman ρ (rho)	0.009	0.117	/	/	/	/	/	/	/	/	/	/	/
	p	0.926	0.245											
PLT	Spearman ρ (rho)	0.012	0.188	0.173	/	/	/	/	/	/	/	/	/	/
	p	0.909	0.062	0.086										
MPV	Spearman ρ (rho)	0.185	0.122	-0.073	-0.33**	/	/	/	/	/	/	/	/	/
	p	0.065	0.225	0.470	0.001									
GLU	Spearman ρ (rho)	-0.053	-0.054	0.416**	-0.007	0.029	/	/	/	/	/	/	/	/
	p	0.597	0.591	<00.001	0.942	0.774								
RBC	Spearman ρ (rho)	0.053	0.082	0.208*	0.015	0.004	-0.040	/	/	/	/	/	/	/
	p	0.597	0.420	0.037	0.883	0.969	0.694							
HGB	Spearman ρ (rho)	0.268**	0.26**	0.049	0.052	0.058	-0.104	0.706**	/	/	/	/	/	/
	p	0.007	0.008	0.625	0.605	0.567	0.305	<00.001						
HCT	Spearman ρ (rho)	0.401**	0.28**	0.132	0.035	0.199*	-0.046	0.673**	0.690**	/	/	/	/	/
	p	<00.001	0.004	0.191	0.728	0.047	0.653	<00.001	<00.001					
MCV	Spearman ρ (rho)	0.407**	0.255*	-0.101	0.096	0.178	-0.041	-0.310**	0.081	0.431**	/	/	/	/
	p	<00.001	0.011	0.315	0.342	0.077	0.687	0.002	0.421	<00.001				
MCH	Spearman ρ (rho)	0.178	0.183	-0.145	0.065	0.072	-0.059	-0.455**	0.207*	-0.039	0.511**	/	/	/
	p	0.077	0.069	0.150	0.520	0.476	0.559	<00.001	0.039	0.699	<00.001			
MCHC	Spearman ρ (rho)	-0.277**	-0.151	-0.094	-0.047	-0.176	-0.021	-0.146	0.152	-0.529**	-0.477**	0.418**	/	/
	p	0.005	0.134	0.354	0.640	0.079	0.836	0.148	0.132	<00.001	<00.001	<00.001		
RDW	Spearman ρ (rho)	0.242*	0.228*	-0.012	0.128	0.499**	-0.023	0.103	0.113	0.124	0.046	0.019	-0.089	/
	p	0.015	0.022	0.907	0.203	<00.001	0.824	0.307	0.263	0.221	0.647	0.850	0.381	
WBC	Spearman ρ (rho)	-0.114	0.061	0.222*	0.560**	-0.277**	0.109	0.017	0.011	0.059	0.114	0.074	-0.042	-0.090
	p	0.259	0.548	0.027	<00.001	0.005	0.280	0.870	0.911	0.561	0.260	0.463	0.681	0.371

Spearman correlation in healthy Bosnian children (Table 7) also showed significant positive correlations between BMI, HGB, HCT, MCV, and age. MCH and erythrocytes showed positive correlations with age as well. PLT showed a significant negative correlation with age in healthy children. HbA1c showed significant negative correlations with MCH and MCHC, and a significant positive correlation with RDW. Other correlations of hematological indices with HbA1c and glucose, in healthy children, were not significant. The number of erythrocytes and white blood cells did not show a significant correlation with HbA1c.

**Table 7:** Spearman correlation for analysed parameters in children with type 1 diabetes mellitus (N=100) All correlations were tested using Spearman test. Spearman ρ (rho), value of Spearman’s rank correlation coefficient, where »-« represents negative correlation, »**« correlation is significant at the 0.01 level, »*« correlation is significant at the 0.05 level. p – significance of correlation between parameters. BMI (kg/m^2^), body mass index; HbA1c (%), hemoglobin A1c; PLT (10^9^/L), platelets; MPV (fL), mean platelet volume; GLU (mmol/L), glucose; RBC (10^12^/L), red blood cells; HGB (g/L), hemoglobin; HCT (%), hematocrit; MCV (fL), mean corpuscular volume; MCH (pg), mean corpuscular hemoglobin; MCHC (g/L), mean corpuscular hemoglobin concentration; RDW (%CV), red blood cell distribution width; WBC (10^12^/L), white blood cells.

		AGE	BMI	HbA1c	PLT	MPV	GLU	RBC	HGB	HCT	MCV	MCH	MCHC	RDW
BMI	Spearman ρ (rho)	.582**	/	/	/	/	/	/	/	/	/	/	/	/
	p	.000												
HbA1c	Spearman ρ (rho)	.009	-.033	/	/	/	/	/	/	/	/	/	/	/
	p	.932	.747											
PLT	Spearman ρ (rho)	.314**	* -.066	.134	/	/	/	/	/	/	/	/	/	/
	p	.001	.516	.182										
MPV	Spearman ρ (rho)	.191	.220*	.137	-.35**	/	/	/	/	/	/	/	/	/
	p	.057	.028	.173	.000									
GLU	Spearman ρ (rho)	.103	-.051	.168	-.033	.040	/	/	/	/	/	/	/	/
	p	.307	.615	.095	.745	.695								
RBC	Spearman ρ (rho)	.220*	.178	.050	.186	-.068	-.112	/	/	/	/	/	/	/
	p	.028	.076	.622	.064	.500	.268							
HGB	Spearman ρ (rho)	.526**	.36**	-.168	-.015	-.008	-.033	.660**	/	/	/	/	/	/
	p	.000	.000	.094	.880	.936	.742	.000						
HCT	Spearman ρ (rho)	.538**	.42**	-.029	.045	-.004	-.050	.740** .	.919**	/	/	/	/	/
	p	.000	.000	.775	.660	.968	.622	.000	.000					
MCV	Spearman ρ (rho)	.496**	.34**	-.067	-.203*	.108	.021	-.317**	.348**	.339**	/	/	/	/
	p	.000	.001	.505	.043	.285	.836	.001	.000	.001				
MCH	Spearman ρ (rho)	.393**	.218*	-.230*	-.218*	.112	.024	-.403**	.337**	.165	.873**	/	/	/
	p	.000	.029	.021	.029	.266	.816	.000	.001	.101	.000			
MCHC	Spearman ρ (rho)	.049	-.133	-.401**	-.154	-.030	.055	-.263**	.112	-.234*	.085	499**	/	/
	p	.629	.189	.000	.127	.767	.584	.008	.269	.019	.402	.000		
RDW	Spearman ρ (rho)	-.006	.039	.304**	.002	.165	.007	.167	-.170	.000	-.173	-.356** -	-.497**	/
	p	.955	.697	.002	.988	.101	.943	.097	.091	.999	.085	.000	.000	
WBC	Spearman ρ (rho)	.071	.103	-.052	.304**	-.132	-.052	0.018	-.004	.016	-.085	-.088 .	.021	.007
	p	.480	.308	.607	.002	.191	.604	.286	.966	.876	.398	.383	.835	.944

## Discussion

Altered levels of many hematological parameters (red blood cells, white blood cells, platelet function) and their corresponding indices (HGB, HCT, MCV, MPV) have been observed in patients with diabetes. However, some authors did not report such differences except for higher WBC count in diabetics. In this study, hematological parameters were tested and compared in a group of children with diabetes and their healthy peers. A careful look at our results shows many changes in the diabetic population, one of them also being white blood cell count which was lower in children with type 1 diabetes mellitus, mean value 6.49×10^12^, compared to their healthy peers (mean 7.44×10^12^ leukocytes, p<0.001).

Observed differences were neither sex nor age affected. Similar results related to the lower number of leukocytes in pathogenesis of type 1 diabetes were reported by Harsunen et al. [12]. The discrepancy in reported leukocyte counts observed in different studies might be explained by their measurements in different stages of diabetes or from the fact that the data are related to different ethnic groups. Therefore, further investigations are required to explain these differences in WBC counts.

From all white blood cells, neutrophils are most involved in the pathogenesis of type 1 diabetes; thus, WBC count alters mainly due to alteration in neutrophil count. Early studies showed that type 1 diabetes patients had higher white blood cells, primarily neutrophil numbers than the controls [13], and increased neutrophil counts were reported to correlate with an augmented cardiovascular complication risk [13]. However, more recent studies showed that circulating neutrophil number and white blood cells consequently were decreased in patients with type 1 diabetes and healthy autoantibody positive subjects, which might be associated with cell specific autoimmunity [14] [12] [23] [24]. Lower Neutrophil count mediates a decrease in total white blood cell count.

Although our research did not include neutrophil counts, WBC data correlates with the second, more recent studies of Valle et al. [14], Harsunen et al. [12], Wang et al. [23], and Battaglia [24]. We found significantly lower WBC values in children with T1DM, compared to healthy controls.

When other hematology data are concerned, descriptive statistics analysis in our study shows a lower number of erythrocytes, lower hematocrit, and hemoglobin levels in patients with type 1 diabetes mellitus when compared to their healthy peers. The Mann-Whitney U test shows a significant difference between healthy group and children with T1DM in number of erythrocytes, hematocrit and hemoglobin values, with p<0.001 for all three parameters analyzed. Interestingly, the number of erythrocytes and the level of hemoglobin were significantly lower in the diabetic female population, whereas MPV was significantly higher when compared to the male diabetic population. Gender differences were not apparent in the healthy population. Hemoglobin, hematocrit, and MCV values also differ by age in both diabetic and healthy children. Mean hemoglobin values in children with type 1 diabetes mellitus in our research were lower when compared to healthy peers, 137.2 vs. 143.4 g/L, respectively. This is in accordance with results reported by Khudhur et al. [25], Mbata et al. [26], and Kothari et al. [27], who reported lower mean values for RBC, HGB, and HCT in diabetics when compared to controls. All of the abovementioned parameters are indicators for the future development of anemia, a common finding in patients with diabetes, particularly in those with nephro pathy and renal impairment as well as retinopathy and macrovascular disease.

When other anemia associated hematological indexes were examined such as MCH and MCHC, differences at the level of different populations as well as gender differences were not evident. However, age differences in values of MCHC and RDW for diabetic, and MCH for healthy children, were evident.

One of the red blood cells indicators often investigated for its association with anemia in diabetic patients is red blood cell distribution with (RDW). Mean RDW values in our study did not differ significantly between healthy and children with type 1 diabetes, 11.81 vs. 11.96%, respectively, with p=0.088 determined by the Man-Whitney. Similar results were reported in numerous other studies [28] [29]. Contrary to these data, Biadgo et al. [20], in a similar comparative study on 296 participants, reported a significant difference in red blood cell distribution width between patients with diabetes and healthy controls. Differences across the study populations, as well as differences in study design and ethnicity, may account for the variability of RDW across studies.

Knowing that diabetes is considered to be a prothrombotic condition where platelets play an active role [30] [31] [32], it was of great interest to examine the differences in platelet size and number between diabetic and healthy populations. In this context, we were especially interested in MPV since this parameter represents a direct marker of platelet function and activity [19]. Several studies have reported higher MPV values in individuals with both T1DM and T2DM, and these changes have been linked to metabolic control of the disease [2] [33]. The average MPV values for healthy Bosnian children were 6.32 fL, while their counterparts with type one diabetes had an average MPV value at 8.32 fL. This difference, as determined by the Mann-Whitney U test, was significant with p<0.001. Similar results as ours were reported in diabetic children by Venkatesh et al. [34], Demirtunc et al. [35], Zuberi et al. [31], Biaggo et al. [20], Winocour et al. [36], and Çoban et al. [37] [38].

Interestingly, Uko et al. [22] observed a significant positive correlation between high PLT and WBC count and raised blood sugar level among the diabetic subjects studied (r=0.52 and 0.45), respectively. Other studies showed positive correlations between WBC and HbA1c, such as a recent meta-analysis of 20 studies, which included 90,000 participants where a positive correlation between increased WBC level and diabetes risk was found [9].

In Sulochana et al. [39] study among the 100 type 2 diabetic patients with increased glycated hemoglobin, total leukocyte count was elevated in about 18 of 46 male and 22 of 54 female patients. In Bosnian children with type 1 diabetes, WBC count showed a significant negative correlation with HbA1c and glucose. In the study by Milosevic et al. [21], no such correlation was reported on patients with type 2 diabetes. Reported results are contradictory and require further attention.

The effects of glucose control and levels of HbA1c (indicators of disease control) on hematological parameters were investigated in this study in three groups of children.

We expected significant alterations in RDW and other RBC parameters reported in other studies [40]. In our study, statistically significant differences between groups with different levels of disease control were observed only for erythrocyte count and hematocrit value (p=0.013 and 0.019, respectively), excluding expected differences in glucose and HbA1c levels. When assessing the correlation between HbA1c and hematological indices in diabetic children and their healthy peers, in Bosnian children, the number of erythrocytes, as well as white blood cells, showed a significant positive correlation with HbA1c and at the same time negative correlation with MCH and MCHC and positive for RDW in healthy children. Interestingly, some [41] authors reported a significant positive correlation between MPV and HbA1c (R=306, p<0.005), while others on non-diabetic subjects observed an inverse correlation between HbA1c and MCV (r=-0.22, p<0.05), MCH (r=-0.30, p<0.05), and MCHC (r=-0.32, p<0.05) [42]. MCV, MCH, and MCHC showed no significant correlation with HbA1c in numerous studies reported [40] [43]. On the contrary, Jaman et al. [44] reported a statistically significant decrease of HCT in diabetic patients (HbA1c 7.5) when compared with non-diabetic patients (HbA1c 6.5). A decrease in the HCT value of diabetic patients may be due to the dehydration and accumulation of protein.

Ulutaş et al. [45] also reported a positive correlation between MPV level and HbA1c level, which is following our results. Hekimsoy et al. [46], have shown the opposite, while Ersoy et al. [47], reported similar MPV values in children diagnosed with T1DM to those in a control group (8.74±0.96 and 8.49±0.66, respectively). These different results in platelet count could be explained by their dependency on several variables, such as mean platelet survival, platelet production rate, and turnover rate in diabetes mellitus. Our study has some limitations related to the effects of the long-term course of the disease, and these limitations have to be overcome in future studies.

## Conclusions

In this study, statistically significant differences in numerous hematological parameters of diabetic Bosnian children were observed when compared to healthy controls. The mean values of erythrocytes, hematocrit, and hemoglobin for diabetic children were found to be within reference ranges but significantly lower than the values of the control group, indicating possible development of anemia in the former group. At the same time, MCV, MCH, and MPV were higher in children with Type 1 diabetes mellitus indicating an alteration in erythrocyte morphology. Hemoglobin, hematocrit, MCV, MCHC, and RDW were age-dependent. Erythrocyte count, hemoglobin, and MCV showed significant gender differences. Age and gender had no effect on MPV, WBC, glucose, or HbA1c levels in children with type 1 diabetes mellitus. The mean value of white blood cells was also found to be lower in diabetic children, supporting a general involvement of the innate immune system in the pathogenesis of type 1 diabetes. Significant differences in mean values of erythrocyte and white blood cells were supported by observed correlations between HbA1c and: WBC and RBC. A significant positive correlation between WBC and HbA1c, as well as RBC and HbA1c, was observed.

Our findings suggest that hematological indices as accessible, simple, inexpensive laboratory parameters could be a useful tool in following up T1DM in children. MPV, in addition to HbA1c, can serve as a prognostic tool for determining the risk of diabetic microvascular and macrovascular complications.

## Acknowledgments

This study was supported by a grant received from the Federal Ministry of Science and Education for the 2017 year awarded to MM. Project title: The importance of determining the parameters of oxidative stress, inflammation, and hemostasis in the early diagnosis of obesity in the pediatric population, number: 05-39-2571-1/17.

## List of abbreviations

BMI, body mass index; CBC, complete blood count; HbA1c, hemoglobin A1c; HCT, hematocrit; HGB, hemoglobin; IQR, interquartile range; MCH, mean corpuscular hemoglobin; MCHC, mean corpuscular hemoglobin concentration; MCV, mean corpuscular volume; MD, medical doctor; MPV, mean platelet volume; PLT, platelets; RBC, red blood cells; RDW, red blood cell distribution width; rpm, revolutions per minute; T1DM, type 1 diabetes mellitus; T2DM, type 2 diabetes mellitus; WBC, white blood cells.

## References

[1] Karvonen M, Viik-Kajander M, Moltchanova E, Libman I, Laporte R, Tuomilehto J, Diabetes Mondiale Diamond Project Group (2000). Incidence of childhood type 1 diabetes worldwide. Diabetes Care.

[2] Korkmaz O (2018). Assessment of the platelet parameters in children with type 1 diabetes mellitus. J Endocrinol Metab.

[3] Lipton R B, Drum M L, Danielson K K, Greeley S A, Bell G I, Hagopian W A (2011). Onset features and subsequent clinical evolution of childhood diabetes over several years. Pediatr Diabetes.

[4] Ersoy M, Elevli M, Ersoy O, et al. (2015). Intima-media thickness and mean platelet volume in children with type 1 diabetes mellitus. Iran J Pediatr.

[5] Nathan D M (1993). Long-term complications of diabetes mellitus. N Engl J Med.

[6] Krishnan S, Short K R (2009). Prevalence and significance of cardiometabolic risk factors in children with type 1 diabetes. J Cardiometab Syndr.

[7] Fendler W, Mlynarski W, Wegner O, Baranowska-Jazwiecka A, Szadkowska A, Tomasik B, Malachowska B (2015). Altered Platelets' morphological parameters in children with type 1 diabetes: A case-control study. BMC Endocr Disord.

[8] Kakouros N, Rade J J, Kourliouros A, Resar J R (2011). Platelet function in patients with diabetes mellitus: From a theoretical to a practical perspective. Int J Endocrinol.

[9] Shukla K D, Chandra P K, Pawah K A (2016). Study of hematological indices in patients with diabetes mellitus and hypertensive diabetes mellitus. International Journal of Medicine Research.

[10] Biadgo B, Melku M, Mekonnen A S, Abebe M M (2016). Hematological indices and their correlation with fasting blood glucose level and anthropometric measurements in type 2 diabetes mellitus patients in Gondar, Northwest Ethiopia. Diabetes Metab Syndr Obes.

[11] Milošević D, Lukić-Panin V (2019). Relationship between hematological parameters and glycemic control in type 2 diabetes mellitus patients. J Med Biochem.

[12] Gkrania-Klotsas E, Ye Z, Cooper A J, et al. (2010). Differential white blood cell count and type 2 diabetes: Systematic review and meta-analysis of cross-sectional and prospective studies. PLoS One.

[13] Singh M, Shin S (2009). Changes in erythrocyte aggregation and deformability in diabetes mellitus. Indian J Exp Biol.

[14] Uko E K, Isaac I Z, Abdulrahaman Y, Adias T C, Sani Y, Shehu R S, Liman H M, Dalltu M K, Mainasara A S (2013). Some haematological parameters in patients with type-1 Diabetes in Sokoto, North Western Nigeria. J Blood Lymph.

[15] 15. American Diabetes Association (2019). Children and adolescents: Standards of medical care in diabetes. Diabetes Care.

[16] Harsunen M H, Puff R, D'orlando O, Giannopoulou E, Lachmann L, Beyerlein A, von Meyer A, Ziegler A G (2013). Reduced blood leukocyte and neutrophil numbers in the pathogenesis of type 1 diabetes. Horm Metab Res.

[17] Huang J, Xiao Y, Xu A, Zhou Z (2016). Neutrophils in type 1 diabetes. J Diabetes Investig.

[18] Valle A, Giamporcaro G M, Scavini M, et al. (2013). Reduction of circulating neutrophils precedes and accompanies type 1 diabetes. Diabetes.

[19] Wang Y, Xiao Y, Zhong L, Ye D, Zhang J, Tu Y, Bornstein S R, Zhou Z, Lam K S L, Xu A (2014). Increased neutrophil elastase and proteinase 3 and augmented NETosis are closely associated with betacell autoimmunity in patients with type 1 diabetes. Diabetes.

[20] Battaglia M (2014). Neutrophils and type 1 autoimmune diabetes. Curr Opin Hematol.

[21] Khudhur K K, Al-Ani M (2019). Hematological parameters in children with type-1 diabetes. Medical Journal of Babylon.

[22] Mbata C A, Adegoke A, Nwagu C, et al. (2015). Some haematological parameters in diabetic patients in Port Harcourt Nigeria. AJMS.

[23] Bokariya P, Kothari R (2012). A comparative study of haematological parameters in type I diabetes mellitus patients & healthy young adolescents. Int J Biol Med Res.

[24] Thomas M C, Macisaac R J, Tsalamandris C, Power D, Jerums G (2002). Unrecognized anemia in patients with diabetes. Diabetes Care.

[25] Oyedemi S O, Yakubu M T, Afolayan A J (2011). Antidiabetic activities of aqueous leaves extract of Leonotis leonurus in streptozotocin induced diabetic rats. J Med Plant Res.

[26] Rampersad M, Thomas S (2004). Anaemia in diabetes. Acta Diabetol.

[27] Stamatakis E, Batty D G, Hamer M, Czernichow S, Kengne A P (2012). Anaemia, haemoglobin level and cause-specific mortality in people with and without diabetes. PLoS One.

[28] Cakir L, Aktas G, Enginyurt O, Cakir S (2014). Mean platelet volume increases in type 2 diabetes mellitus independent of HbA1c level. Acta Medica Mediterranea.

[29] Dada O A, Uche E, Akinbami A, et al. (2014). The relationship between red blood cell distribution width and blood pressure in patients with type 2 diabetes mellitus in Lagos. Nigeria. J Blood Medicine.

[30] Sharpe P C, Trinick T (1993). Mean platelet volume in diabetes mellitus. QJM.

[31] Kodiatte T A, Manikyam U K, Rao S B, Jagadish T M, Reddy M, Lingaiah H K M, Lakshmaiah V (2012). Mean platelet volume in type 2 diabetes mellitus. J Lab Physicians.

[32] Zuberi B F, Akhtar N, Afsar S (2008). Comparison of mean platelet volume in patients with diabetes mellitus, impaired fasting glucose and nondiabetic subjects. Singapore Med J.

[33] Ates O, Kiki I, Bilen H, et al. (2009). Association of mean platelet volume with the degree of retinopathy in patients with diabetes mellitus. Eur J Gen Med.

[34] Venkatesh V, Kumar R, Varma D K, Bhatia P, Yadav J, Dayal D (2018). Changes in platelet morphology indices in relation to duration of disease and glycemic control in children with type 1 diabetes mellitus. J Diabetes Complications.

[35] Demirtunc R, Duman D, Basar M, Bilgi M, Teomete M, Garip T (2009). The relationship between glycemic control and platelet activity in type 2 diabetes mellitus. J Diabetes Complications.

[36] Winocour P D (1994). Platelets, vascular disease, and diabetes mellitus. Can J Physiol Pharmacol.

[37] Coban E, Kucuktag S, Basyigit S (2007). Platelet activation in subjects with impaired glucose tolerance. Platelets.

[38] Coban E, Bostan F, Ozdogan M (2006). The mean platelet volume in subjects with impaired fasting glucose. Platelets.

[39] Ulutas K T, Dokuyucu R, Sefil F, Yengil E, Sumbul A T, Rizaoglu H, et al. (2014). Evaluation of mean platelet volume in patients with type 2 diabetes mellitus and blood glucose regulation: A marker for atherosclerosis?. Int J Clin Exp Med.

[40] Hekimsoy Z, Payzin B, Örnek T, Kandoğan G (2004). Mean platelet volume in Type 2 diabetic patients. J Diabetes Complications.

[41] Ersoy M, Selcuk D H N, Elevli M N, Ersoy O, Civilibal M (2015). Aortic intima-media thickness and mean platelet volume in children with type 1 diabetes mellitus. Iran J Pediatr.

[42] Sulochana S, Viswanath A (2017). Correlation of total leucocyte count and differential leucocyte count in relation to glycated haemoglobin in type 2 diabetes. Int J Health Sci Res.

[43] Bhutto A R, Abbasi A, Abro A H (2019). Correlation of hemoglobin A1c with red cell width distribution and other parameters of red blood cells in type II diabetes mellitus. Cureus.

[44] Jaman M S, Rahman M S, Swarna R R, Mahato J, Miah M M, et al. (2018). Diabetes and red blood cell parameters. Ann Clin Endocrinol Metabol.

[45] Kurt H, Demirkiran D (2016). Changing of hemoglobin A1c affects mean platelet volume in type-2 diabetes mellitus. Ulutas Med J.

[46] Rafal W, Mojs E, Stanislawska-Kubiak M, Wojciak, (2014). The occurrence of iron-deficiency anemia in children with type 1 diabetes. J Investig Med.

[47] Hardikar P S, Joshi S M, Bhat D S, Raut D A, Katre P A, Lubree H G, Jere A, Pandit A N, Fall C H D, Yajnik C S (2012). Spuriously high prevalence of prediabetes diagnosed by HbA1c in young Indians partly explained by hematological factors and iron deficiency anemia. Diabetes Care.

[48] Koga M, Morita S, Saito H, Mukai M, Kasayama S (2007). Association of erythrocyte indices with glycated haemoglobin in pre-menopausal women. Diabet Med.

